# Sex-dependent effects of chronic exercise on cognitive flexibility but not hippocampal Bdnf in aging mice

**DOI:** 10.1042/NS20210053

**Published:** 2022-01-05

**Authors:** Annabel K. Short, Viet Bui, Isabel C. Zbukvic, Anthony J. Hannan, Terence Y. Pang, Jee Hyun Kim

**Affiliations:** 1 Florey Department of Neuroscience and Mental Health, University of Melbourne, Parkville, VIC, Australia; 2 Mental Health Theme, The Florey Institute of Neuroscience and Mental Health, Parkville, VIC, Australia; 3 IMPACT – The Institute for Mental and Physical Health and Clinical Translation, School of Medicine, Deakin University, Geelong, VIC, Australia

**Keywords:** aging, bdnf, cognition, fear, neurotrophic factors, sex

## Abstract

Cognitive impairments associated with advanced age involve alterations in the hippocampus that changes with experience throughout life. The hippocampus is critical for cognitive flexibility involved with extinction and reinstatement of conditioned fear. It is widely accepted that regular exercise can be beneficial for hippocampal function. Therefore, we asked whether chronic voluntary exercise in middle-aged mice can improve extinction and/or reinstatement of conditioned fear compared with standard-housing. Eight-month-old male and female C57Bl/6J mice had access to a running wheel or remained in standard-housing until 11 months of age. Alongside control standard-housed young adult (3-month-old) mice, they received tone–footshock pairings, which were subsequently extinguished with tone-alone presentations the next day. Half of the mice then received a reminder in the form of a single footshock. Male and female 11-month-old mice housed in standard conditions exhibited impaired reinstatement compared with young adult mice. However, for males that had access to a running wheel from 8 months of age, the reminder treatment rescued reinstatement ability. This was not observed in females. Additionally, exercise during middle age in both sexes increased expression of brain-derived neurotrophic factor (*Bdnf*) mRNA in the hippocampus, specifically exon 4 mRNA. These results show that, at least for males, physical exercise is beneficial for reducing age-related decline in cognitive abilities. Despite not affecting reinstatement, exercise also increased *Bdnf* gene expression in the female hippocampus, which could potentially benefit other forms of hippocampus-dependent cognition.

## Introduction

People with a physically active lifestyle are known to have some protection against the effects of normal aging on cognition. The correlation between aerobic exercise and improved cognition is strong, and exercise appears to be effective across the lifespan [1–3]. This has significant implications for older adults who face heightened risk of cognitive decline.

The effects of exercise on cognition has been linked with hippocampal function [2,4]. Although many studies highlight changes in the hippocampus volume and connectivity as the neural correlate for exercise effects in humans [1–3,5], the molecular correlates are poorly understood. Rodent models have been useful in this regard, with chronic exercise shown to alleviate the decrease in hippocampal neurogenesis and synaptic plasticity in aging rodents [6,7]. Importantly, the hippocampus is sexually dimorphic in rodents and humans [8]. Indeed, sex differences in hippocampus-dependent learning have been widely reported in rodents and humans [9–11]. However, findings on sex differences in cognitive decline with age is inconsistent in humans [12–14]. In rodents, there are very few reports of sex differences in non-pathological cognitive decline with age [15]. Sex differences are observed following exercise in hippocampus-dependent tasks. A meta-analysis in humans reported females having greater cognitive improvements than males after aerobic training [16]. A meta-analysis in rodents describes no sex differences in spatial tasks following aerobic training, but greater improvements in non-spatial cognitive tasks in males [16]. Sex-specific effects on cognition and underlying neurobiology clearly need further examination to understand lifestyle factors associated with healthy aging.

Cognitive flexibility is one of the most impaired facets of intelligence due to age [17]. Cognitive flexibility is the ability to adapt to a changing environment and is often tested using reversal or set shifting tasks [18,19]. In our study, cognitive flexibility was assessed using reinstatement following extinction of conditioned fear. Mice were first conditioned with a tone conditioned stimulus (CS) that was paired with a footshock unconditioned stimulus (US), which led to freezing to the CS as a measure of emotional memory of the conditioning session (CS-US). Then the CS was presented repeatedly without the US, which decreases the freezing to the CS to form the extinction memory (CS-no US). When tested in the same context as extinction, the CS-no US memory is typically retrieved, evidenced by low levels of freezing. However, a single reminder footshock can facilitate the retrieval of the conditioning memory and lead to high freezing (i.e., reinstatement). Taken together, reinstatement can test cognitive flexibility because it requires flexible retrieval of the extinction versus conditioning memory [6,20,21]. While it is not the typical model to study cognitive flexibility, it is widely agreed that expression of reinstatement requires a complex understanding of environmental cues to be flexible in the choices of responses, and deficit in such flexibility may be related to persistence of fear observed in anxiety disorders [22–25]. Consistent with these ideas, hippocampal lesions impair reinstatement [26,27]. Reinstatement is particularly appropriate for this study because previous studies have found that it is sensitive to age and sex effects [28–31].

The aim of the present study was to examine whether chronic exercise in middle-age can rescue potential cognitive flexibility impairments in aging male and female mice. Eight-month-old mice had access to running wheels in their home cage or standard-housed for 3 months and were tested at 11 months of age for reinstatement of conditioned fear. This testing age shows natural cognitive decline [32,33], while avoiding the onset of reproductive senescence in female mice occurring at 12–15 months of age [34] that may reduce sex differences. We also tested reinstatement in 3-month-old mice as young adults [35,36] to provide a baseline because they reliably display reinstatement of extinguished fear [37]. Notably, all the mice from the present study were obtained at 8 weeks of age and were housed in the same facility until the end of the study, an important detail given that most previous studies in aging mice used retired breeders with unknown history. In addition, we examined hippocampal brain-derived neurotrophic factor (*Bdnf*) gene expression of these mice to see if the beneficial effects of exercise are reflected at a molecular level. Increased hippocampal *Bdnf* expression in freely exercising rodents is a well-established molecular correlate for the exercise-associated benefits on brain and behavior [38–40]. In particular, *Bdnf* exon 4 transcript expression has been implicated in extinction of conditioned fear in rodents [41,42]. We hypothesized that any age-related impairment of reinstatement and hippocampal *Bdnf* expression would be reversed by voluntary exercise during middle age. Based on a meta-analysis in mice [16], we also anticipated that exercise effects would be greater in males than females.

## Materials and methods

### Animals

**Figure 1:**
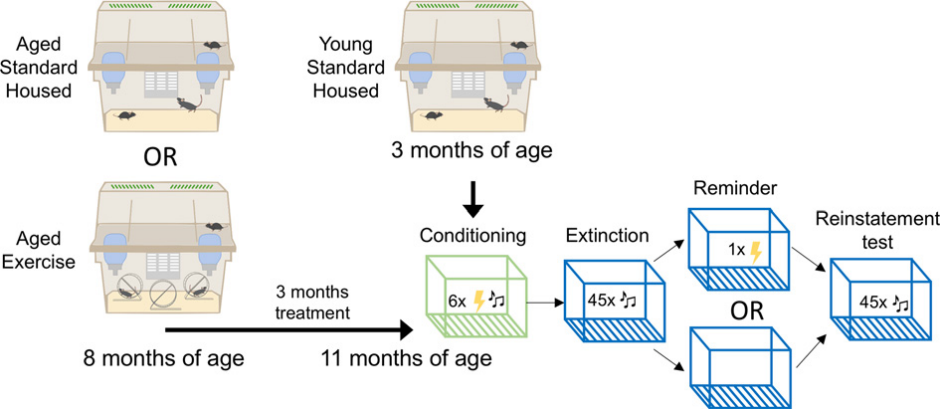
Experimental design C57Bl/6J mice at 8 weeks of age were purchased and group housed (2–3 mice, males and females separated) in double decker cages (floor area: 2527.5 cm^2^). Aged groups were aged to 8 months then assigned to either Standard-Housing or Exercise conditions. Exercising animals had access to one running wheel per mouse (2–3 wheels placed in housing; 12 cm diameter with floor area of 66 cm^2^) for 3 months until behavioral testing commenced at 11 months of age. Exercise wheels were removed the day before behavioral testing. Young adult controls were housed as Standard-Housing aging mice and were tested at 3 months of age concurrently with the aged mice. Behavioral protocol occurred over 4 days, with mice receiving either a shock or no shock to test reinstatement of extinguished fear.

C57Bl/6J mice at 8 weeks of age were purchased from the Animal Resources Centre (Murdoch, WA, Australia). All animals were group housed (2–3 mice, males and females separated) in large cages dedicated to mice experimentation (40.3 × 46.2 × 40.4 cm, floor area 2527.5 cm^2^, GR1800 double decker, Tecniplast, Australia) with temperature and humidity at 22°C and 45%, respectively. Cages were lined with sawdust and two tissues provided for nesting with food and water *ad libitum*. Mice were maintained on a 12-h light/dark cycle (lights on at 07:00) and bedding changed weekly. Aged groups were aged to 8 months then assigned to either Standard-Housing or Exercise conditions (Figure 1). Exercising animals had access to one running wheel per mouse (2–3 wheels placed in housing; 12 cm diameter with wheel floor area of 66 cm^2^) for 3 months until behavioral testing commenced at 11 months of age. Exercise wheels were removed the day before behavioral testing. Young adult controls were housed similar to Standard-Housing aging mice and were tested at 3 months of age concurrently with the aged mice (Figure 1). Therefore, there were three treatment conditions in the present study. All procedures were approved by the Florey Institute of Neuroscience and Mental Health Animal Ethics Committee. Due to the long duration of the present study (∼12 months), target sample size was calculated *a priori* using G*Power [43] based on between-subjects design with 12 groups at reinstatement test (3 Treatment × 2 Sex × 2 Reminder conditions), large effect size (Cohen’s f = 0.35), a = 0.05 and power (1-b) = 80%. Effect size estimation is based on our previous work in reinstatement [28,37]. This yielded a total N=83. Therefore, 84 mice were purchased and used in the present study.

### Apparatus

Behavioral chambers were rectangular (31.8 × 25.4 × 26.7 cm) with grid floors with 36 rods (3.2 mm), equipped with a VideoFreeze system (Med Associates, VT, U.S.A.). A constant-current shock generator delivered electric shocks to the floor of the chambers as required. A programmable tone generator, speaker and sound calibration package was used to deliver tones (volume: 80 dB; frequency: 5000 Hz). In order to create two different contexts for conditioning versus extinction, the chambers differed in appearance as described previously [44]. Animals were randomly assigned to different starting contexts.

### Conditioning

Mice at either 3 or 11 months of age were placed in the chamber. Baseline freezing was measured for 2 min. All animals then received six tone–footshock pairings. Each pairing comprised 10 s tone co-terminating with a 1-s shock (0.7 mA). Inter-trial intervals (ITIs) ranged from 85 to 135 s (110 s average).

### Extinction

The day following conditioning, mice were tested for their tone memory by being placed in a second chamber of differing appearance to the conditioning chamber to create a separate context from conditioning. Baseline freezing was measured for 2 min, then 10-s tone was presented 45-times in the absence of the shock (ITI = 10 s).

### Reminder

The day following extinction, the mice were further divided into two Reminder groups per treatment. One group (Reminder) received a single reminder shock (0.7 mA, 1 s) without a tone in the extinction context, and the other group (No Reminder) was placed in the extinction context but did not receive any shock or tone.

### Reinstatement test

One day after reminder, the mice were tested for reinstatement in the extinction context. Baseline freezing was measured for 2 min, then 10-s tone was presented 45-times in the absence of the shock (ITI = 10 s).

### Culling

Following reinstatement test, all mice were killed by pentobarbital (Virbac, Australia) intraperitoneal injection (100 mg/kg), except for 21 mice that were kept for hippocampal *Bdnf* quantification (see below).

### RNA extraction

To avoid immediate effects of behavior on RNA levels, 21 mice were killed 1 week following the final behavioral session by cervical dislocation and whole hippocampi were microdissected from males (young standard *n*=4; aged standard *n*=4; aged running *n*=3) and females (young standard *n*=4; aged standard *n*=3; aged running *n*=3). RNA was extracted using QIAGEN RNeasy Mini Extraction Kit as per manufacturer’s instructions (QIAGEN, VIC, Australia). Tissue samples were disrupted using a Diagenode Biorupter (UCD-300; Life Research, VIC, Australia) in the QIAGEN lysis buffer. On-column DNAse1 treatment was performed, and RNA was eluted in 50 μl of RNase-free water. RNA concentrations and purity were determined using Nanodrop spectrograph (2000c Thermo Scientific, DE, U.S.A.). Samples were then stored at −80°C until required.

### Reverse transcription

Thawed RNA (1000 ng) was reverse transcribed using Superscript VILO cDNA synthesis kit (Invitrogen, Life Technologies, Australia). Reverse-transcription PCR was performed in a thermal cycler (Takara Shuzo, Japan) using 1× cycle of the following program: 25°C for 10 min, 48°C for 30 min and 95°C for 5 min. Samples were then stored at −20°C.

### Real-time quantitative PCR

Levels of gene expression in the tissue was quantified using real-time quantitative PCR (qPCR) using the Viia 7 Real-Time PCR system (Applied Biosystems, CA, U.S.A.). Reactions were made using: SYBR Green, 10 μl (S4438, Sigma–Aldrich, Australia), ROX reference dye 0.2 μl (12223-012, Invitrogen), forward and reverse primers (20 μM) 0.5–1.5 μl each, cDNA 50 ng in 5 μl, DNAse-free H_2_O up to 20 μl. Cyclophillin was used as the endogenous control. Primer sequences (Sigma–Aldrich) are listed as following:

Cyclophillin forward 5′ CCCACCGTGTTCTTCGACA 3′,
Reverse 5′ CCAGTGCTCAGAGCTCGAAA 3′;
*Bdnf* total forward 5′ GCGCCCATGAAAGAAGTAAA 3′,
Reverse 5′ TCGTCAGACCTCTCGAACCT 3′;
*Bdnf* Exon 4 forward 5′ CAGAGCAGCTGCCTTGATGTT 3′,
Reverse 5′ GCCTTGTCCGTGGACGTTTA 3′.

Optimal primer dilutions and amplification efficiencies had previously been determined by TYP and AKS. PCRs were run on the following program: 50°C for 2 min, 95°C for 10 min, followed by 40× cycles of 95°C for 15 s and 60°C for 1 min. The expression levels of the target genes were determined using comparative *C*_t_ (ΔΔ*C*_t_) method and normalized to the mean expression of the young adult male control group.

### Statistical analysis

Statistics were computed using SPSS statistics version 22.0 (IBM, NY, U.S.A.). For analysis of the conditioning data, freezing data were based on the first 9 s of each CS and to exclude the effects of the electric shock on movement. For extinction at testing, freezing was scored over all 10 s of each CS [45,46]. To reduce pseudoreplication, 45 trials were collapsed into nine blocks of five tones for extinction and reinstatement, consistent with a previous study [46]. Analyses of variance (ANOVAs) were used analyzed potential effects of Sex, Treatment, Reminder and interactions among these factors on freezing levels and levels of mRNA expression. Main effects were followed up with Tukey’s *post hoc t*ests, while significant interactions were followed with Fisher’s LSD post hoc tests.

At conditioning, there was a significant effect of Sex on baseline freezing (F_(1,72)_ = 4.05, *P*<0.05) with males freezing more than females, there were no other effects or interactions (*P*-values >0.05). At extinction, there were no effects or interactions (*P*-values >0.05) in baseline freezing. At reinstatement test, there was an effect of Reminder on baseline freezing (F_(1,72)_ = 28.38, *P*<0.001) with mice that received the reminder footshock freezing more than those that did not, there were no other effects or interactions (*P*-values >0.05). To control these differences at baseline, CS-elicited freezing for each behavioral session was analyzed with analyses of co-variance (ANCOVAs) with baseline freezing levels as a co-variate as described in previous studies [30,47]. However, the results of ANCOVA did not differ from results of ANOVA without baseline as a co-variate, therefore, we report the CS-elicited freezing results of ANOVA below.

## Results

### Running reduced weight in aging mice

**Figure 2:**
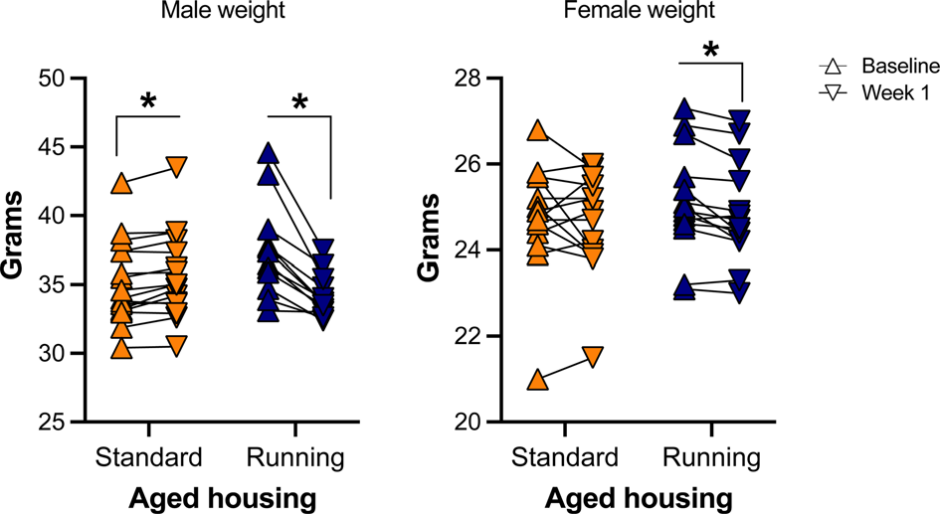
Weight gain at baseline and 1 week after running wheels were placed in aged running groups Male (aged standard = 16; aged running = 12) and female (aged standard = 14; aged running = 14) mice with running wheels placed for 1 week lost weight, while aged standard-housed male mice gained weight (*P<0.05 post-hoc effect of Time following a significant Time × Sex × Exercise interaction). Aged standard-housed female mice did not change their weight.

As an indirect way of ensuring that aged mice were running, we recorded individual weights only in aging mice right before (baseline) and 1 week after the insertion of wheels. Repeated-measures (RMs) ANOVA of weight change across those two timepoints showed that there was a Time × Exercise × Sex interaction (F _(1, 52)_ = 37.52, *P*<0.001), indicating that weight change across time in either standard or running wheel housed mice depended on sex. To understand this three-way interaction, we followed up with paired *t* tests per group with Bonferroni’s corrections for multiple testing. There were significant effects of Time indicating increased weight for aged standard-housed males (*P*<0.005), but decreased weight in aged running males (*P*<0.001) and females (*P*<0.005). Aged standard-housed females did not show any weight change (*P*>0.05). These results show that in standard-housed aging mice, weight increase was only observed in males, while running reduced weight in both sexes (Figure 2).

### Aging impairs cognitive flexibility which in males is rescued with exercise

**Figure 3:**
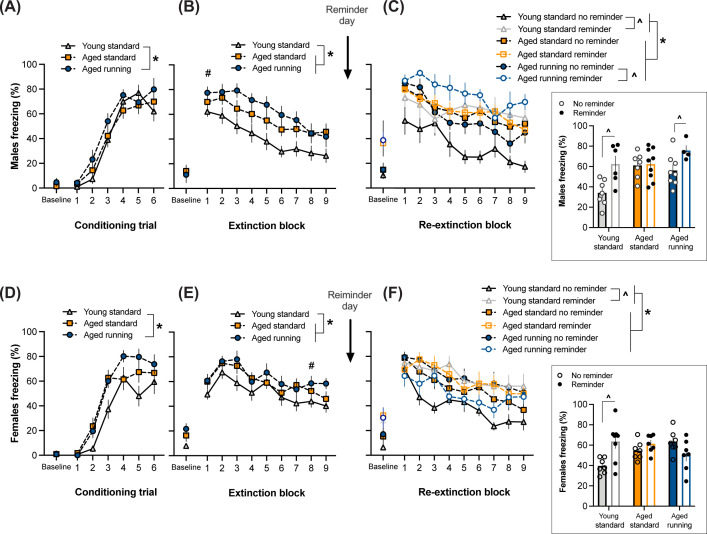
Effect of age and exercise on fear conditioning, extinction and reinstatement test (mean ± SEM) There were no effects of Reminder condition before the reminder session (i.e., conditioning and extinction), hence these sessions represent data pooled across Reminder condition. (**A**) While all mice increased their CS-elicited freezing across conditioning trials, aged mice with access to running wheels freeze more when compared with young mice sexed as males (A) and females (**D**) (*P<0.05 main effect of Treatment across entire session with post-hoc Tukey’s comparisons). (**B**) Aging elevates CS-elicited freezing levels during extinction in males (B) and females (**E**) (*P<0.05 main effect of Treatment across entire session with significant post-hoc differences). In addition, males overall display greater fear retrieval in the beginning of extinction followed by a steeper extinction curve (i.e., accelerated extinction learning) compared to females (^#^*P*<0.05 indicated in the sex that displayed more freezing at that particular block, Sex × Block interaction with significant post-hoc effect of Sex), however, there was no sex difference by the final extinction block. (**C**,**F**) Test data of 45 tone presentations with 5 tones per block. Boxed inset represents CS-elicited freezing when averaged across all 45 tones. Aged groups showed elevated overall CS-elicited freezing than young mice (*P<0.05 main effect of Treatment across entire session with significant post-hoc differences). (C) In males, reinstatement is impaired in aged animals living in standard-housing, which is rescued by access to running wheels. (F) In females, reinstatement is impaired in aged mice regardless of access to running wheels (^*P*<0.05 Treatment × Sex × Reminder interaction with significant post-hoc effect of Reminder). Conditioning and extinction: males (young standard = 13; aged standard = 16; aged running = 12) and females (young standard = 15; aged standard = 14; aged running = 14). Re-extinction/reinstatement test: males (young standard no shock = 7, young standard shock = 6; aged standard no shock = 7, aged standard shock = 9; aged running no shock = 8; aged running shock = 4) and females (young standard no shock = 7, young standard shock = 8; aged standard no shock = 7, aged standard shock = 7; aged running no shock = 7, aged running shock = 7).

RM ANOVA of CS-elicited freezing during conditioning revealed main effects of Trial (F_(5,360)_ = 147.99, *P*<0.001) and Treatment (F_(2,72)_ = 4.26, *P*<0.05). *Post hoc* tests revealed that aged mice with access to running wheels froze more than young mice (*P*<0.01). There were no effects of Sex, Reminder or interactions between any of the factors (*P*-values >0.05), confirming no pre-existing differences between the Reminder condition before the reinstatement/reminder session in the present study. Hence Figure 3A,D show pooled data of both Reminder conditions. These results indicate that while all mice increased their CS-elicited freezing across conditioning trials, aged mice with access to running wheels freeze more when compared with young mice regardless of Sex.

RM ANOVA of CS-elicited freezing during extinction (Figure 3B,E) revealed effects of trial Block (F_(8,576)_ = 35.91, *P*<0.001) and Treatment (F_(2,72)_ = 6.70, *P*=0.002). *Post hoc* tests revealed that both groups of aged mice froze more than young mice (*P*-values <0.05). There was a Block × Sex interaction (F_(8,576)_ = 5.13, *P*<0.001) with *post hoc* tests showing males freezing more than females at block 1 (*P*<0.001), then females freezing more than males at block 8 (*P*<0.05), and no other blocks showing sex effects (*P*-values >0.05). In case the differences in the rate of extinction between males and females were suggestive of a lack of within-session extinction in females, we also conducted an RM ANOVA of extinction blocks only in females, which showed a significant effect of Block (F_(8,336)_ = 14.09, *P*<0.001), clearly indicating that extinction occurred in all female groups. There were no other effects or interactions during extinction (*P*-values >0.05), again highlighting that there were no pre-existing differences between the Reminder condition before the reinstatement/reminder session in the present study. Hence Figure 3B,E show pooled data of both Reminder conditions. These results suggest that aging elevates CS-elicited freezing levels during extinction in males and females. In addition, males overall display greater fear retrieval in the beginning of extinction followed by a steeper extinction curve (i.e., accelerated extinction learning) compared with females.

Half of the mice received a reminder session 1 day after extinction (Figure 1). One day after this session, the mice were tested for reinstatement (Figure 3C,F). RM ANOVA of CS-elicited freezing revealed effects of trial Block (F_(8,576)_ = 40.09, *P*<0.001) and Block × Reminder interaction (F_(8,576)_ = 2.54, *P*<0.01). Within-subjects factor Block did not interact with any other factors (*P*-values >0.05). Block × Reminder interaction was clearly driven by the strong main effect of Reminder (F_(1,72)_ = 13.27, *P*<0.001). There was also a significant main effect of Treatment (F_(2,72)_ = 5.41, *P*<0.01), with *post hoc* tests showing that young adult mice overall froze less than both groups of aged mice (*P*-values <0.05). There also were interactions of Treatment × Reminder (F_(2,72)_ = 6.02, *P*<0.005) and Treatment × Reminder × Sex (F_(2,72)_ = 3.30, *P*<0.05). There were no other interactions (*P*-values >0.05). The three-way interaction was followed up with *post hoc* tests, which showed that in males, the reminder effectively reinstated extinguished fear in young adult (*P*<0.01) and aged running (*P*<0.05) mice but not in standard-housed aged mice (*P*>0.05). In females, only young adults showed reinstatement (*P*<0.01), with no reinstatement in both aged groups (*P*-values >0.05). Taken together, aging decreased cognitive flexibility in both sexes, which was rescued with exercise in males but not in females.

### Spontaneous recovery

**Figure 4:**
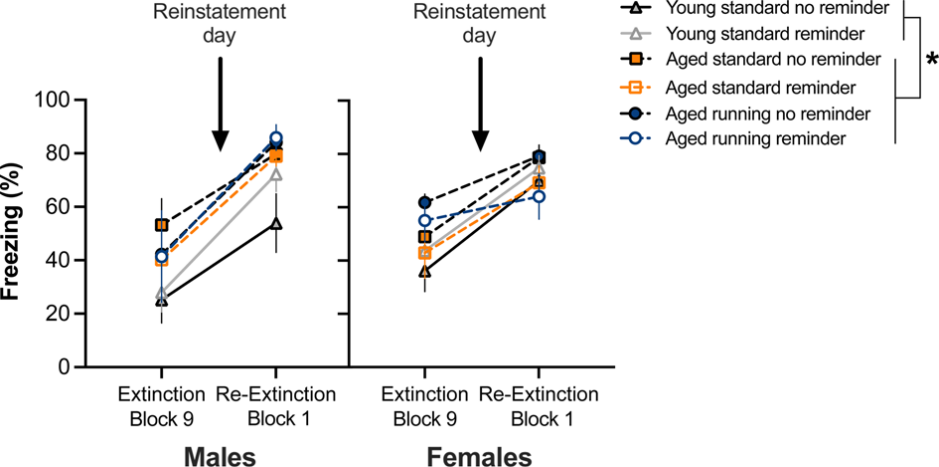
CS-elicited freezing at extinction block 9 and re-extinction block 1 (mean ± SEM) First block of re-extinction showed significantly increased freezing compared with the last block of extinction (*P*<0.05). Consistent with the ongoing Treatment effect at extinction and re-extinction, young mice froze less overall than aging mice (*P<0.05 main effect of Treatment with significant post-hoc differences). Reminder and Sex had no effects. Males (young standard no shock = 7, young standard shock = 6; aged standard no shock = 7, aged standard shock = 9; aged running no shock = 8; aged running shock = 4) and females (young standard no shock = 7, young standard shock = 8; aged standard no shock = 7, aged standard shock = 7; aged running no shock = 7, aged running shock = 7).

Upon inspection of Figure 3C,F, it appeared that all mice spontaneously recovered regardless of Reminder for the first block of re-extinction compared with the last block of extinction, which suggests a dissociation between spontaneous recovery and reinstatement. Hence, we used RM ANOVA to compare CS-elicited freezing during those blocks (Figure 4). This revealed effects of Block (F_(1,72)_ = 158.67, *P*<0.001), Treatment (F_(2,72)_ = 5.37, *P*<0.01) and Block × Sex interaction (F_(1,72)_ = 6.91, *P*<0.05). There were no other effects or interactions (*P*-values >0.05), indicating that Reminder had no effect before it occurred (extinction last block), and at the first block of re-extinction. *Post hoc* tests for the main effect of Treatment showed that young adult mice overall froze less than both groups of aged mice (*P*-values <0.05), which is consistent with the overall Treatment effect seen across extinction and re-extinction reported above. Block × Sex interaction was followed-up with testing Sex differences per Block, which did not reveal any statistically significant differences (*P*-values >0.05). These results indicate that all mice displayed spontaneous recovery at the initial re-extinction session, and the effect of reminder to induce reinstatement is observed with more presentations of the CS at re-extinction.

### Exercise up-regulates hippocampal Bdnf mRNA levels in male and female mice

**Figure 5:**
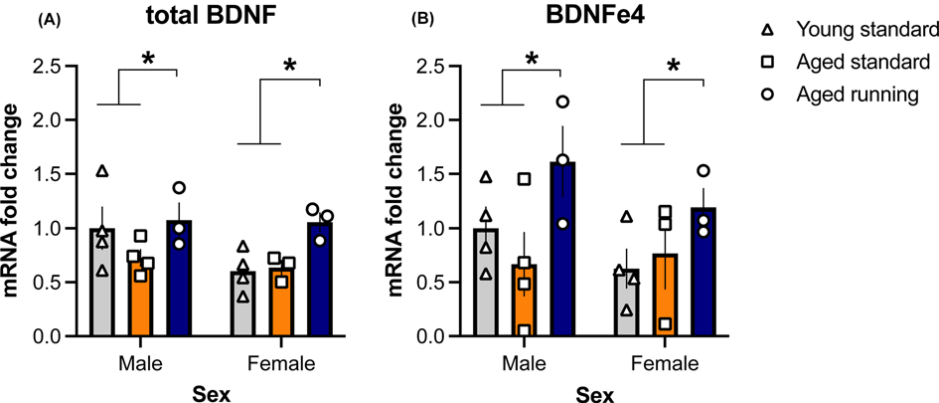
Effect of age and exercise on hippocampal *Bdnf* mRNA expression (Mean ± SEM) (**A**) Total *Bdnf* transcript expression in the hippocampus was significantly increased following 3 months of exercise compared with standard-housing in aged and young mice (*P<0.05 main effect of Treatment with post-hoc Tukey’s comparisons). (**B**) *Bdnf* exon 4 transcript expression in the hippocampus was significantly increased following 3 months of exercise compared with standard-housing in young and aged mice (*P<0.05 main effect of Treatment with post-hoc Tukey’s comparisons). *n*=3–4 per group.

One-week following the final behavioral session, whole hippocampi were microdissected and analyzed. While there was no significant Sex × Treatment interaction (F_(2,15)_ = 2.218, *P*=0.143) nor effect of Sex (F_(1,15)_ = 4.415, *P*=0.053) for total *Bdnf* (Figure 5A), there was an overall effect of Treatment (F_(2,15)_ = 7.473, *P*<0.05). Tukey’s post hoc comparisons revealed that aged running mice had higher levels of total *Bdnf* compared with the aged and young standard mice (*P*-values <0.05). Similarly, for *Bdnf* exon 4 (Figure 5B), there was no Sex × Treatment interaction (F_(2,15)_ = 0.978, *P*=0.399) nor effect of Sex (F_(1,15)_ = 1.908, *P*=0.187), but there was a significant effect of Treatment (F_(2,15)_ = 6.04, *P*<0.05). Tukey’s post hoc comparisons revealed increased *Bdnf* exon 4 mRNA expression in aged running animals compared with young standard and aged standard-housed animals (*P*-values <0.05).

## Discussion

We showed that (1) aging impairs reinstatement in both sexes; (2) aerobic exercise during middle age can restore reinstatement in males but not in females; and (3) exercise during middle age increases expression of total *Bdnf* and *Bdnf* exon 4 mRNA in both males and females. Secondary findings include combined effects of aging and running increases freezing during conditioning, and aging increases overall freezing during extinction and test. There was a subtle effect of males showing accelerated extinction acquisition compared with females, however, they were not different by end of extinction.

### Exercise, sex and age effects on cognitive flexibility and hippocampal Bdnf

The present study observed significant impairments in cognitive flexibility as measured by reinstatement of extinguished fear in mice at 11 months of age compared to mice at 3 months of age. A decrease in functional connectivity between the prefrontal cortex (PFC) and the hippocampus with age that may predict cognitive performance has been reported [48]. Such connectivity is critical for the relapse of extinguished fear [49,50], hence a decreased connectivity in these extinction-related pathways would explain the present finding of age-associated impairment in fear reinstatement.

Strikingly, 3 months of exercise during middle age restored the age-related reinstatement deficit in males but not females. Sex-specific effects of exercise on fear extinction have been reported in young adult rats, in which exercise during extinction consolidation significantly improved extinction in males but had no effect on females [51]. Similarly, chronic voluntary exercise improves extinction retention in adolescent male but not in female rats [52]. These findings highlight that the sex-specific findings of exercise on reinstatement of extinguished fear is likely not age-specific, that exercise at any age may result in sex differences in extinction of conditioned fear. There is growing evidence for sex-specificity in how previous experiences impact cognitive flexibility, which may relate to how experiences may affect the PFC more in males than females [53].

The outcomes of the present study also add to the emerging literature on the sex-specific effects on cognition, where previously in humans it has been reported that females having greater cognitive improvements than males following exercise [16], here we report opposing effects in aged mice. Other than the species differences, the level of exercise in mouse versus human studies may explain the contrasting findings. The systematic review in humans included any exercise intervention that occurred at least once a week [16], whereas mice in the present study had continuous access to exercise wheels. Thus, it is highly likely that exercise sessions in mice were much more frequent than the ones in humans, which may have affected the outcomes on cognition. Another variable is age. The female mice in the present study were pre- or peri-menopausal, whereas females were well past their menopause in aging human studies of cognition following exercise [16].

In the present study, hippocampal *Bdnf* does not appear to drive the protective effect of exercise in aging males. It is highly unlikely the mRNA findings are due to behavioral procedures because increases in hippocampal BDNF mRNA following fear conditioning are only maintained up to 24 h [54], and the tissues were collected 1 week after final behavioral test in the present study. Also, any effect of fear conditioning would be the same across all the groups because all the animals were treated identically. Including exon 4, the *Bdnf* gene contains multiple exons which undergo alternative splicing to create multiple exon-specific transcripts of *Bdnf* [55,56]. In addition to *Bdnf* exon 4 being required for the extinction of conditioned fear in rodents [41,42], it is also an important regulator of the activity-dependent effects of BDNF protein. The exon 4 promoter contains a CRE-binding site that is thought to be responsible for the cyclic adenosine monophosphate (cAMP) initiated transcription of BDNF important for experience-dependent plasticity [57,58]. In young adult rodents, both males [38] and females [39] have increased levels of *Bdnf* mRNA within the hippocampus following voluntary wheel running.

Given the sex-specific effects of exercise on reinstatement of conditioned fear in aging mice, the lack of sex differences in *Bdnf* expression was surprising. *Bdnf* is an important regulator of exercise-induced hippocampal neurogenesis [59]. Neurogenesis following exercise can be sex-specific [8]. In contrast, a recent study in adolescent rats showed that neurogenesis following exercise can significantly increase without affecting extinction recall in group housed male and female rats [52]. In that study, sex-specific neurogenesis was only observed when rats were single-housed, which was not the case in the present study. Clearly, the relationship among exercise, learning and memory, neurogenesis and *Bdnf* expression warrant further investigation. Though we describe no sex differences in *Bdnf* mRNA expression, previous work reported voluntary access to running wheels at 2 months of age for 5 months had increased hippocampal levels of mature BDNF protein in males only, while females had higher sedentary protein expression [60]. In that study, both males and females had increases in *Bdnf* exon 4 mRNA following 5 months of exercise beginning in early adulthood as we have observed, however only males had increased total *Bdnf* mRNA. While expression of *Bdnf* has been found to decrease with age in rats [61] and in humans [62], a correlative decline with cognition has only been reported for females [16,62].

The present study focused on the hippocampus because of its importance in reinstatement. While there was an increase in hippocampal *Bdnf* following exercise in both sexes, in females there was no improvement in cognitive flexibility. This suggests that either increased *Bdnf* is not sufficient to restore age-related deficits in cognitive flexibility, or expression of hippocampal *Bdnf* is non-essential for fear reinstatement in females. Other brain regions such as the PFC, wherein dendritic spines density is increased by exercise [63], may also explain the sex-specific effects of exercise on cognitive flexibility but not hippocampal *Bdnf*. Our purposeful inclusion of females into the present study has highlighted the complexity of the relationship of *Bdnf* and cognitive flexibility.

### Aging effects on enhanced conditioned fear expression

Across both sexes, aged animals with access to running wheels froze more overall during fear acquisition. During extinction and re-extinction, aged animals regardless of exercise had increased freezing. One interpretation is an overall impairment of hippocampal function in aged animals that led to impaired extinction recall because hippocampus inactivation can impair extinction recall [64,65]. Alternatively, these findings suggest an age-related enhancement of cued fear memory consolidation because the increased CS-elicited freezing at re-extinction in aging groups has already manifested during the initial extinction. Aging and exercise together may also facilitate acquisition of cued fear memory, but increased freezing at extinction the next day in aging groups does not appear to be affected by exercise. A similar increase in freezing with cued fear in aged mice has been reported [66]. The amygdala is a central brain region regulating the acquisition and consolidation of cued fear [67–69]. While aged-related changes in amygdala structure and volume are poorly understood, there may be an overall loss of functional connectivity of the amygdala with the broader cortical regions [70]. However, the mechanisms whereby loss of innervation of the amygdala could result in increased conditioned fear consolidation remains to be investigated.

### Spontaneous recovery and reinstatement

Statistically comparing the last block of extinction to the first block of re-extinction indicated that all mice spontaneously recovered regardless of the reminder condition in the present study. This is interesting and suggests a dissociation between spontaneous recovery and reinstatement processes. Such dissociation between spontaneous recovery and reinstatement suggests that cognitive flexibility in the form of spontaneous recovery is intact in aging mice.

While these two forms of relapse following extinction of conditioned fear, as well as renewal, are often discussed together [22,71,72], there is some evidence that the three are dissociated processes. For example, fornix lesions can abolish reinstatement but not spontaneous recovery and renewal [27]. Conversely, hippocampus lesions can abolish renewal but not reinstatement [26]. In another study, the levels of renewal were similar while the spontaneous recovery levels were altered when fear conditioning and extinction intervals were manipulated [73]. The present results showing how spontaneous recovery emerges early in re-extinction whereas reinstatement is observed during overall re-extinction further add to the evidence that the widely studied relapse processes following extinction of conditioned fear may have different underlying mechanisms.

## Limitations

A limitation of the present study is that estrous cycle and estrogen levels of the females were not monitored. CS-elicited freezing levels during extinction were nuanced based on sex. Males maximally froze from block 1 (mean 69.68), which was significantly higher than freezing levels in females (mean 56.56). Females appeared to reach maximal freezing in blocks 2 and 3. In addition, females froze more than males at block 8 (mean 51.45 and 39.01, respectively) but there were no sex differences by block 9. These results indicate that extinction acquisition may be temporarily delayed in females compared with males. A similar finding was reported previously in rats, in which females undergoing estrous phases associated with high estradiol and progesterone levels (i.e., metestrus, diestrus and proestrus) showed delayed extinction compared with males [74]. In that study, such effects were persistent and lasted until the end of extinction. In the present study, females and males froze at similar levels by the end of extinction. This may be due to mixed number of estrus phases represented in the females in the present study and/or hormones having reduced influenced in the aging females in the present study. Estrous cycle has been shown to affect fear conditioning and extinction in female rodents and humans [75,76], with recent evidence that estrous cycle and related sex hormones drive extinction differences between male and female rats [74,75]. The low variability in the female behavioral data in the present study suggests that it is unlikely that estrous cycle is influencing our results, with female mice at 11 months of age considered a middle age with lengthened but still regular estrous cycling [34].

In addition, running activity was not monitored, so it is unclear if the exercise levels were equivalent between males and females. Future work should aim to quantify running, although we have shown previously that extinction recall outcomes were unaffected by running levels, whether matched or different between sexes in adolescent rats [52]. In another previous study, we observed no sex differences in running wheel activity over 1 month in group housed mice aged 3, 8 or 13 months [33], which suggests that it is unlikely that males and females ran differently in the present study. In addition, BDNF levels were induced to similar extents in males and females, suggesting that the running stimulus was sufficient to activate exercise-associated molecular signaling programs in the hippocampus to affect behavior. However, it is important to note that the analyses of *Bdnf* were not powered to identify small effect sizes in the present study.

## Conclusions

Overall, our study suggests that exercise during middle age reduces the extent of cognitive impairments in males but not females. The benefits of exercise described here highlight the importance of tailoring exercise recommendations/expectations to the individual, with sex being an important consideration. Reinstatement of conditioned fear appears to be a helpful rodent model to delineate sex and age effects on cognition, and we hope that our observations will facilitate further studies to understand the molecular correlates of exercise benefits on cognitive flexibility.

## Data Availability

Data are available upon request.

## Open Access

Open access for this article was enabled by the participation of University Of Melbourne in an all-inclusive *Read & Publish* pilot with Portland Press and the Biochemical Society under a transformative agreement with CAUL.

## Abbreviations

ANCOVA: analysis of co-variance
ANOVA: analysis of variance
Bdnf: brain-derived neurotrophic factor
CS: conditioned stimulus
ITI: inter-trial interval
PFC: prefrontal cortex
qPCR: real-time quantitative polymerase chain reaction
RM: repeated-measure
US: unconditioned stimulus
